# Comparison of Several Models for Calculating Ozone Stomatal Fluxes on a Mediterranean Wheat Cultivar (*Triticum durum* Desf. cv. Camacho)

**DOI:** 10.1100/tsw.2007.243

**Published:** 2007-10-05

**Authors:** Daniel de la Torre Llorente

**Affiliations:** University of Salamanca, Ecology Department, Pharmacy Building, Campus Miguel de Unamuno, Salamanca 37008, Spain

**Keywords:** wheat, Triticum durum, ozone stomatal fluxes, 3-D architecture model, models

## Abstract

Ozone stomatal fluxes were modeled for a 3-year period following different approaches for a commercial variety of durum wheat (*Triticum durum* Desf. cv. Camacho) at the phenological stage of anthesis. All models performed in the same range, although not all of them afforded equally significant results. Nevertheless, all of them suggest that stomatal conductance would account for the main percentage of ozone deposition fluxes. A new modeling approach was tested, based on a 3-D architectural model of the wheat canopy, and fairly accurate results were obtained. Plant species-specific measurements, as well as measurements of stomatal conductance and environmental parameters, were required. The method proposed for calculating ozone stomatal fluxes (FO_3_3-D_) from experimental g_s_ data and modeling them as a function of certain environmental parameters in conjunction with the use of the YPLANT model seems to be adequate, providing realistic estimates of the canopy FO_3_3-D_, integrating and not neglecting the contribution of the lower leaves with respect to the flag leaf, although a further development of this model is needed.

## INTRODUCTION

The critical level of ozone for the protection of vegetation used during the 1990s — and currently in use, although its use is changing towards cumulative absorption indexes — in the UNECE (United Nations Economic Commission for Europe) framework is the AOT40 (the accumulated exposure of $O_3$ over 40 nl $l^{-1}$ along a given time period when the photosynthetic photon flux density, PPFD > 50 W $m^{-2}$)[13,23,37]. Nevertheless, there is scientific evidence to suggest that the effects of ozone are much more strongly linked to ozone absorption by plants than merely to exposure to ozone[5,18,24,26,29,35]. This is based on the consideration that the uptake of phytotoxic ozone by leaves is strongly controlled by stomatal conductance and that its uptake via the cuticle is negligible[25]. Thus, current critical ozone levels are based on cumulated ozone concentrations, vapor pressure deficit (VPD)–modified cumulated ozone concentrations, and cumulative ozone uptakes, for a given period, depending on the type of vegetation[38]; the last one just for some agricultural crops, i.e., wheat and potato, and provisionally for sensitive forest trees, i.e., beech and birch. The method for calculating cumulative ozone uptake accepted by the UNECE-EMEP (European Monitoring and Evaluation Programme) was developed by Emberson et al.[10], based on previous work by Jarvis[22], and its application has been checked by several authors in wheat[8,35], potato[35], clover[29], and forest trees[24,44], among other studies.

Cumulative ozone uptake is calculated as the sum of ozone stomatal fluxes in a given period, which, as well as depending on the ozone concentration, depends on the particular species and even on the variety considered. It also depends on environmental factors, the sensitivity of the species to changes in environmental factors, the phenological stage of the plant, the health status of the plant, etc. In other words, many different factors control stomatal uptake. Nevertheless, in a healthy plant of any given species and/or variety, the main factors on which stomatal conductance and ozone stomatal fluxes are dependent are environmental parameters, such as temperature, radiation, VPD, and soil water potential (SWP).

In the present study, within the framework of UNECE-EMEP, ozone stomatal fluxes were modeled for a durum wheat cultivar (*Triticum durum* Desf. cv. Camacho) along the phenological stage of anthesis, where the maximum stomatal conductance occurs[9,34,35]. Ozone stomatal fluxes were modeled following several approaches.

## MATERIALS AND METHODS

The ozone stomatal fluxes over a durum wheat canopy (*T. durum* Desf. cv. Camacho) were assessed using several approaches. The study was carried out at "El Encín" (40º31'15'' N, 3º17'27'' W), an experimental field that belongs to the IMIDRA (Institute for Research and Rural Development on Agriculture of Madrid), in Alcalá de Henares (Madrid, Spain), over three consecutive years (2000–2002), following the development of the phenological cycle of the cultivar.

### Observational Data Collection

#### Plant Material and Agronomic Practices

The Camacho cultivar was selected based on a previous experiment addressing the sensitivity of 11 Spanish varieties (data not shown) and because it is representative of Mediterranean nonirrigated crops. The crop plot dimensions were 60 × 60 m (3,600 $m^2$). Sowing density was 200 seeds $m^{-2}$. The cultivar was fertilized following a ratio of 8N:15P:8K and was treated with herbicide (Roundup: N-P-methyl-glycine, $C_3H_8NO_5P$; MONSANTO) prior to sowing. No irrigation was performed, per the usual agricultural practices in the area.

#### Stomatal Conductance and Leaf Area Index Measurements

Stomatal conductance to water vapor ($g_s$, mol $H_2O$ $m^{-2}$ $s^{-1}$) was assessed using portable photosynthesis and gas exchange analyzer systems (Li-Cor 6200 in years 2000 and 2001, and Li-Cor 6400 in years 2001 and 2002; Li-Cor, Lincoln, NE). The measurements were performed on all the green leaves of the plants, at maximum exposure to solar radiation, from 10:00 to 18:00 h GMT, randomly choosing plants that showed a similar growth development at different plot locations and avoiding the border effect. The leaves were numbered from emergence until senescence; thus, the first leaf and that closest to the ground was leaf number one and the last leaf and farthest from the ground was leaf number seven, or the flag leaf (highest in position). At the same time, for each stomatal conductance measurement, PAR (photosynthetically active radiation), leaf temperature (T), and relative humidity (RH) were assessed employing the gas exchange analyzer systems; T and RH were employed to obtain the VPD, following the Goff-Gratch[15] method.

Leaf area index measurements (LAI: $m^2$ leaf area/$m^2$ projected area) were taken with a Li-Cor LAI-2000 analyzer. These measurements were used to convert the stomatal conductance measurements per total leaf area into stomatal conductance per sunlit leaf area, and thus correct the shading effect on the stomatal conductance measurements. It was assumed that the sunlit leaf area/total leaf area ratio followed the same relationship as $LAI_{exp}/LAI_{global}$[7,36], where

$$LAI_{exp} = 1.95\ (1 - e^{-0.7\ LAI}) \qquad (1)$$

is the LAI for leaves exposed to solar radiation.

During clear skies, $LAI_{exp}$ is split into an index for leaves exposed to both direct and diffuse radiation ($LAI_{global}$) or to diffuse only ($LAI_{shade}$)[21]:

$$LAI_{global} = 1 - e^{(-LAI_{exp}/2\cos\theta)})\ 2\cos\theta, \qquad (2)$$

$$LAI_{shade} = (LAI_{exp} - LAI_{global}) \qquad (3)$$

($\theta$ = zenith). Thus, stomatal conductance to water vapor was converted multiplying $g_s$ by $LAI_{exp}/LAI_{global}$.

Soil water humidity was assessed with a tensiometer (Delta T, HH2) at four depths (10, 20, 30, and 40 cm). The probes measured continuously from the flag leaf emergence until senescence.

Measurements were taken at the phenological stage of anthesis, corresponding to a decimal code of 60 to 69[45], since this has been shown to be the most sensitive period for ozone uptake by wheat[2,32,33].

## *Environmental Parameters*

Meteorological data were recorded using three meteorological towers located inside the crop area. One of the towers harbored a conventional anemometer (THIES), a radiation sensor (Kipp & Zonen, mod. CNR1), and a hygrothermothermistor (THIES) for the measurement of temperature and RH, all located at a height of 10 m. Ozone concentrations were analyzed using an automatic monitor (Dasibi Env. Corp. model 1003-RS); the air probe was at a height of 2.5 m above ground level in the second tower. The third tower was equipped with a sonic anemometer (GILL Instruments, mod. Research) at 2.5 m above ground level. All data were recorded automatically with a data logger (Geonica Meteodata 1256C) that afforded mean recordings every 10 min. Ozone concentrations recorded at 2.5-m height were recalculated as ozone concentrations at canopy level using the model developed by Pleijel[31], although no significant differences were found (data not shown).

## *Leaf Levels Grouping as a Function of Stomatal Conductance ($g_s$)*

Prior to use of the stomatal conductance model, an analysis of the covariance (ANCOVA) in observed stomatal conductance was performed, considering the leaf position at the canopy as the fixed factor and PAR as the covariant or independent factor of the mobile mean (STATISTICA 5.0, Statsoft, Inc., 1996). Planned comparisons were obtained for the comparison of leaf $g_s$. The following assumptions were tested:

- Normal distribution of the dependent and covariant variables, by means of the Pearson test ($\chi^2$), comparing observed and expected frequencies
- Homogeneity of variances, by means of the Levene test

- Parallelism or possible interaction of the dependent variable with the covariant, by means of the Fischer-Snedecor test
- Independence of means and variances of the dependent factor, by means of a correlation test

In the event of any of the assumptions not being confirmed, a variable transformation was performed. The more used variable transformations were the logarithmic transformation of [variable + 1] and the box-cox transformation.

## Models Development

### *Modeling Stomatal Conductance as a Function of Environmental Parameters*

Stomatal conductance ($g_s$) was modeled using multiple regressions between the experimental $g_s$ and some environmental parameters (STATISTICA 5.0, Statsoft, Inc., 1996) in order to explain $g_s$ as a function of he environmental parameters, measured in parallel with the gas exchange analyzer, that must modulate it. Once the logarithmic-type relationship for $g_s$ vs. PAR[42] had been obtained, certain environmental parameters other than the PAR, such as temperature, RH, or VPD, were considered in order to explain the maximum variance in $g_s$ in the multiple regression model. Independent models were built for each leaf group with homogeneous $g_s$ values (see above).

In order to assign the most appropriate PAR value to each leaf group in the canopy, the attenuation of PAR due to leaf shading was estimated at each leaf group height for the year 2000; the ratio of the PAR measured at each leaf group height (h) regarding the maximum PAR was calculated, and a simple regression was found for the PAR attenuation ratio vs. height. For 2001 and 2002, an improvement was introduced to estimate the PAR at every leaf height; namely, a 3-D plant architectural model, YPLANT, which simulates plant aerial architecture and estimates the effective PAR at each leaf height, taking into account the intra- and interplant shading at different canopy levels[28,39,40,41]. The YPLANT model does not calculate the PAR as a light extinction coefficient model; based on the simulation of the plant canopy, it calculates the effective PAR at every leaf height.

The YPLANT model demands certain plant input parameters, such as leaf area, leaf length, leaf inclination angle, leaf orientation, leaf height at the shoot knot, plant height, and shoot diameter at each leaf height, as well as some other parameters, such as day and month, latitude, and the solar constant. Previous to the aplication of the YPLANT model, certain green leaf types were defined for every leaf height; every leaf type at each leaf height was structured in 1-, 2-, or 3-length sections, depending on how many changes existed in the leaf inclination angle. For this purpose, eight to 10 representative samples of leaf type for every leaf height were taken.

The main output parameters were total leaf area, leaf “sunlit” area (the leaf area exposed to direct radiation), and the effective PAR at each leaf height, that will be used in the stomatal conductance calculation as a function of the environmental parameters, using the multiple regressions of $g_s$ vs. PAR and VPD or RH.

### *Stomatal Conductance Multiplicative Model*

Stomatal conductance was also modeled following the current EMEP-CLRTAP (Convention on Long Range Transboundary Air Pollution) multiplicative model for calculating $g_s$[10,38], based on the multiplicative model previously developed by Jarvis[22]. A new environmental parameter-algorithm was included for ozone ($g_{O3}$), and the one for the SWP was replaced by relative soil humidity (SH) at a depth of 30 cm (the most significant in a previous study at 20-, 30-, and 40-cm depth, data not shown) because SWP data were lacking; thus, the $g_s$ parameterization would be:

$$g_s = g_{max}\, g_{phen}\, g_{PAR}\, \{(g_{min},(g_{temp}\, g_{VPD}\, g_{SH}\, g_{O3}\,)\} \qquad (4)$$

where each $g_i$ is the $g_s$ parameter-algorithm for each environmental factor considered.

## Ozone Deposition Fluxes

The measurement of total ozone deposition fluxes (stomatal and nonstomatal) over the wheat canopy was performed in 2000 (see above). A fast-response ozone analyzer (LOZ-3, Scintrex Unisearch) was placed inside a sheltered box at the bottom of the tower. The analyzer was calibrated before and after short measurement periods (2–3 days). The required Fetch conditions were taken into account[19,30]. Sample frequency for eddy correlation technique corresponded to the fast-response ozone analyzer (10 Hz). The instrumental response time of the ozone analyzer, including time residence of air inside the 2-m long Teflon sampling line, was calculated to correlate simultaneous vertical wind component and ozone concentration signals, and was fixed at 2.4 s. The chosen average time for flux calculation was 10 min, and ozone detrending was not applied to raw data. Fluxes were corrected by two-axis rotation to minimize aerodynamic effects[20].

In order to be able to validate the ozone stomatal fluxes modeled ($FO_{3\_3\text{-}D}$) as a function of the experimental stomatal conductance, besides the measurements of ozone deposition fluxes and the calculation of ozone stomatal fluxes based on the multiplicative model, the ozone deposition fluxes were also modeled following stomatal resistance ($r_s$)-based models: Baldocchi et al.[4], Coyle[7,12], Erisman et al.[11], Grünhage and Haenel[17], Hicks et al.[21], and Wesley[43]. All these $r_s$-based models calculate $r_s$ from meteorological data as solar radiation and temperature, employing the adequate algorithms. Baldocchi et al. calculate the effect of PAR over the canopy stomatal conductance ($G_{SPAR}$) according to the fractions of sunny and shaded leaf area, and according to the photon flux density expressed as incident PAR over the leaves, where $G_{S(PAR)}$ is corrected by several modulating factors as temperature (g(T)), VPD (g(D)), SWP ($g(\psi)$), and relative diffusivity of contaminant (ozone) ($D_i$) respect to the vapor of water diffusivity ($D_v$), ($D_v/D_i$). Erisman et al. calculate the stomatal resistance employing an algorithm that derives from Baldocchi et al.[4] and that uses global radiation, surface temperature, and internal resistance (depending on season and land use, 120 s $m^{-1}$ in our case). The main differences of Coyle's model from the other models are: (1) it differentiates between stomatal resistance for sunny sky and stomatal resistance for cloudy sky; (2) for cloudy sky stomatal resistance calculation, it uses $LAI_{exp}$, i.e., LAI for those leaves exposed to sun radiation; (3) $LAI_{exp}$ is divided into $LAI_{global}$ for leaves exposed to both direct and diffuse radiation, and $LAI_{dif}$ for leaves exposed just to diffuse radiation[21]. All models calculate the ozone deposition fluxes at canopy level, being completely comparable.

## Calculation of Ozone Stomatal Fluxes ($FO_3$)

It is well known that ozone stomatal uptake is not only a function of the ozone concentration and ozone stomatal conductance (i.e., stomatal resistance), but also of mesophyll resistance, cuticle resistance, and soil resistance. Nevertheless, it was assumed that calculation of ozone stomatal uptake only as a function of the ozone concentration and the ozone stomatal conductance would be a good approach. This assumption was based on Erisman et al.[11], who considered mesophyll resistance to be negligible as compared to stomatal resistance, and according to Grandjean-Grimm and Fuhrer[16], who considered cuticle resistance to be insignificant as compared to stomatal resistance, and who considered soil resistance to be constant, around 500 s $m^{-1}$, which is negligible in comparison with stomatal resistance.

In fact, there are other studies that do take soil resistance into account[1,14], but in order to simplify the present study, and based on the UNECE directive[38], the first assumption was preferred.

Ozone stomatal conductance ($g_s$ ($O_3$)) was calculated by multiplying the hourly mean values of $g_s$ by the molecular diffusivity of ozone to water vapor ($Dr = D_{O3}/D_{H20}$), which according to Nobel[27] is 0.613.

This value was used for calculating the ozone stomatal conductance, following the Ozone Mapping Manual[38]:

$$g_s\,(O_3) = g_s\,D_r \tag{5}$$

Hourly mean $FO_3$ for the canopy were calculated following the method described in Bermejo et al.[6] as the product of hourly mean ozone concentrations ($[O_3]$) at canopy height multiplied by hourly mean ozone stomatal conductance ($g_s\,(O_3)$), and the fluxes were corrected by the LAI-shading correction factor $LAI_{exp}/LAI_{global}$:

$$FO_3\,(\text{nmol m}^{-2}\,\text{s}^{-1}) = g_s\,(O3)[O_3]\,(LAI_{exp}/LAI_{global}), \tag{6}$$

where $g_s$: mol $m^{-2}$ $s^{-1}$; $[O_3]$: nl $l^{-1}$.

The unit usually employed for ozone deposition fluxes is ppb m $s^{-1}$ and, hence, the same unit was employed for the calculation of $FO_3$ in order to be able to compare the fluxes (1 nmol $m^{-2}$ $s^{-1}$ = 0.024 ppb m $s^{-1}$).

The boundary layer resistance ($r_b$) was ignored in the $FO_3$ calculation, following the UNECE directives.

The summed $FO_3$ for all the plant leaves would provide a total $FO_3$ for the canopy that would overestimate the canopy mean ozone deposition flux due to the added contribution of all the different leaves. Thus, in order to be able to compare the modeled $FO_3$ as a function of the experimental $g_s$ to the ozone deposition fluxes, a weighted mean of $FO_3$ was obtained; the mean $FO_3$ for every leaf group was divided by the total $FO_3$ for the whole plant (the summed $FO_3$ for all the plant leaves), obtaining the weighting factor for that leaf group (w.f); then, the weighted mean of $FO_3$ would be the sum of the products of $FO_3$ for every leaf group by its corresponding w.f:

$$FO_{3\_weighed\ mean} = \Sigma\,(FO_{3\_i} * w.f_i)\,, \tag{7}$$

where “i” is the leaf group number.

As stated earlier, there were no significant differences between the ozone concentration at the measuring height and the ozone concentration at the leaf canopy. Although it is certain that there is a gradient of the ozone concentration inside the canopy, the same assumption of no significant differences was assumed for the gradient of the ozone concentration inside the canopy, as the canopy height is only about 50–100 cm, compared to the measuring height of 2.5 m.

## RESULTS

## Observational Data

Mean stomatal conductances and mean environmental parameters are summarized for the anthesis period of each year (Table 1).

### *Leaf Level Grouping as a Function of Stomatal Conductance*

The mean leaf stomatal conductances for each year are summarized in Table 2. The replicates for the leaf stomatal conductances were taken in different days along anthesis for each year; besides, each replicate is the mean value of three measurements in the same leaf. The leaf level groups that resulted from the planned comparisons by means of an ANCOVA analysis of those $g_s$ are shown in Table 3.

**TABLE 1**
**Mean Stomatal Conductance ($g_s$) and Environmental Parameters**

| Parameter | 2000 | 2001 | 2002 |
|---|---|---|---|
| $g_s$ (mmol $m^{-2}$ $s^{-1}$) | 396 | 155 | 126 |
| PAR (μmol $m^{-2}$ $s^{-1}$) | 1534 | 1638 | 1513 |
| T (ºC) | 18 | 23 | 20 |
| RH (%) | 46 | 44 | 42 |
| VPD (kPa) | 2.2 | 1.6 | 1.55 |
| LAI | 3.39 | 2.82 | 1.36 |
| $O_3$ (nl $l^{-1}$) | 35 | 38 | 40 |

*Note*: All mean values are for 24-h cycles, except for $g_s$ and PAR, whose mean values are for daylight hours.

**TABLE 2**
**Mean Stomatal Conductances ($g_s$, mmol $m^{-2}$ $s^{-1}$) for Each Level of Green Leaf and Every Year**

| Year | $g_3$ | $g_4$ | $g_5$ | $g_6$ | $g_7$ |
|---|---|---|---|---|---|
| 2000 | 320 ± 50; n = 9 | 410 ± 30; n = 16 | 370 ± 30; n = 17 | 390 ± 40; n = 15 | 490 ± 30; n = 9 |
| 2001 | 120 ± 30; n = 9 | 130 ± 30; n = 9 | 120 ± 20; n = 7 | 250 ± 30; n = 6 | 280 ± 20; n = 8 |
| 2002 | 84 ± 20; n = 8 | 94 ± 20; n = 9 | 140 ± 50; n = 11 | 160 ± 20; n = 12 | 150 ± 20; n = 12 |

*Note*: $g_s$ are numbered from leaf three to seven (flag leaf), the green leaves at anthesis. Standard deviations (SD, ±) are indicated.

**TABLE 3**
**Significant Planned Comparisons from the ANCOVA Analysis for $g_s$ and Resulting Leaf Groups**

| Year | Groups | *p* Value |
|---|---|---|
| 2000 | 3 vs. 4–6 | <0.01 |
| | 4–6 vs. 7 | <0.01 |
| 2001 | 3–5 vs. 6–7 | <0.01 |
| 2002 | 3–4 vs. 5–7 | <0.05 |

## YPLANT Model Development

### *Stomatal Conductance Modeling as a Function of Environmental Parameters*

For the data from 2000, the effective PAR to each leaf group was assigned by estimating the PAR attenuation as a function of leaf height at the shoot knot (discussed earlier), resulting in the following equation:

$$\text{PAR attenuation (\%)} = 0.973 - 0.012\ h;\ R = -0.73;\ R^2 = 0.53\ ;\ p < 0.001;\ n = 134 \qquad (8)$$

where h is the leaf height at the shoot knot.

For the data from 2001 and 2002, the estimation of the effective PAR was improved by means of the 3-D aerial plant architecture model, YPLANT[28,39,40,41], which provides a much more realistic estimation of PAR. The optimized size for the wheat crop plot in the model was four plant lanes by 12, or 16 different individual-type plants per lane, respectively, for each year. For 2001, plant density was 95 plants $m^{-2}$; the mean distance between plant lanes was 224 mm and, hence, the plot area was 0.5053 $m^2$ with 48 plants in it. For 2002, plant density was 64 plants $m^{-2}$; the mean distance between plant lanes was 70 mm and, hence, the plot area was 1.2544 $m^2$ with 64 plants in it. As a result of this modeling, a 3-D canopy plant architecture model like the one shown in Fig. 1 was obtained, where the intra- and interplant shading can be observed. The model estimates the effective PAR at each leaf height, integrating the shade due to the surrounding leaves. This effective PAR at each leaf height is employed in the calculation of the stomatal conductance as a function of the environmental parameters.

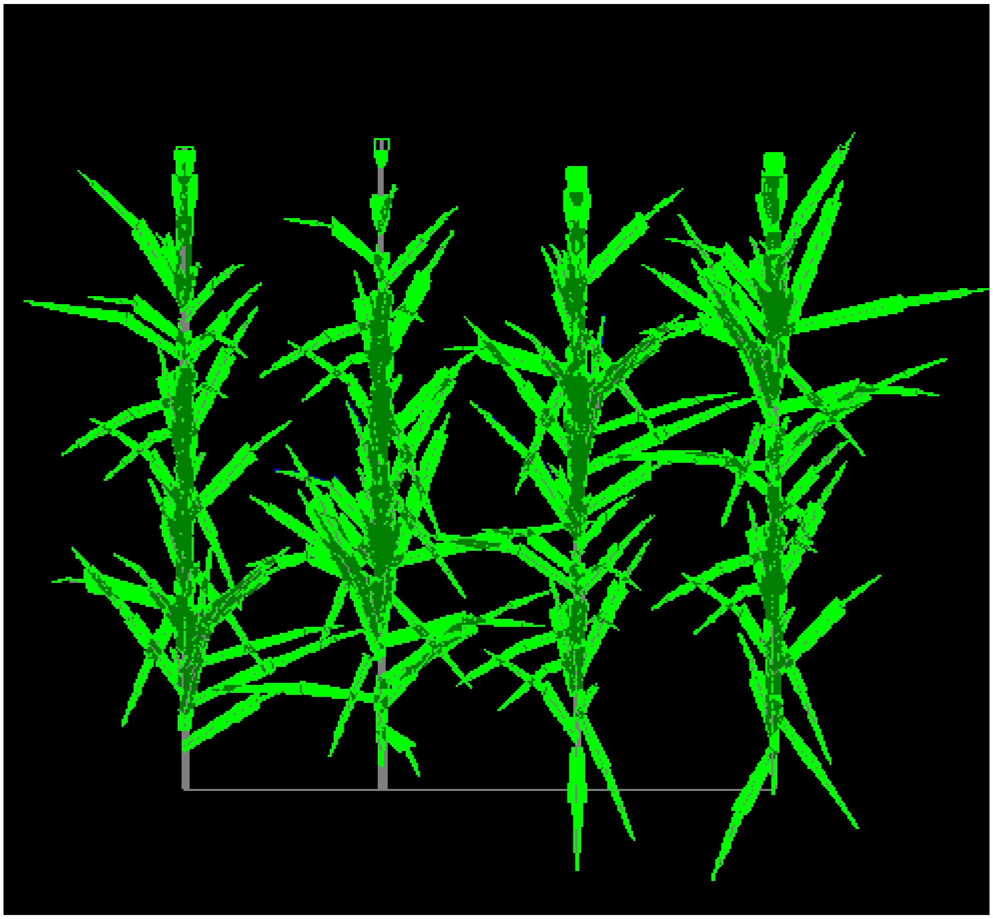

**FIGURE 1.** Wheat 3-D aerial architecture model plot using the YPLANT model[28].

The correlations found for the different years and leaf level groups obtained (see above) are summarized in Table 4.

**TABLE 4**
**Multiple Regressions for $g_s$ as a Function of PAR and VPD**

| Year | Leaf Group | Coefficients | | | $R^2$ | *p Value* | n |
|---|---|---|---|---|---|---|---|
| | | PAR | VPD | Independent | | | |
| 2000 | 3 | 6.73 $e^{-4}$ | –0.467 | 0.0130 | 0.73 | <0.001 | 9 |
| | 4–6 | 0.0147 | –0.665 | 0.0103 | 0.88 | <0.001 | 51 |
| | 7 | 0.0387 | –0.670 | 0.0779 | 0.68 | <0.001 | 9 |
| 2001 | 3–5 | 0.027 | –0.078 | 0.15 | 0.85 | <0.001 | 22 |
| | 6–7 | 0.312 | –0.101 | 0.168 | 0.85 | <0.001 | 7 |
| 2002 | 3–4 | 4.2 $e^{-5}$ | –0.022 | 0.0355 | 0.77 | <0.01 | 10 |
| | 5–7 | 1.6 $e^{-4}$ | –0.025 | 0.2814 | 0.37 | <0.01 | 35 |

*Note:* PAR coefficients are for log(PAR) in 2000 and 2001, where variable transformation was needed due to nonlinearity of $g_s$ vs PAR. Independent coefficient refers to the regression coefficient that is not multiplied by PAR or by VPD.

## Models Comparison

### *Stomatal Conductance Multiplicative Model*

The experimental environmental parameters-algorithms found are shown in Table 5. Maximum and minimum experimental $g_s$ are shown in Table 6.

**TABLE 5**
**Environmental Parameters-Algorithms for the Multiplicative Model**

| Parameter | $g_{PAR}$ | $g_{VPD}$ | $g_T$ | $g_{phen}$ | $g_{O_3}$ | $g_{SH}$ |
|---|---|---|---|---|---|---|
| Algorithm | $1 - e^{(-0.003\ PAR)}$ | $1/(1+(VPD/6)^9)$ | $1/(1+(T/40)^9)$ | $1/(1+(tt/1662)^9)$ | $1/(1+([O3]/65)^9)$ | $1 - e^{(-50\ SH + 6.5)}$ |

*Note:* Algorithms taken from Danielsson et al.[8]. Algorithms coefficients are experimental from 2000, 2001, and 2002 data, considering all leaves (677 data).

**TABLE 6**
**Maximum and Minimum**
**$g_s$ (mmol $m^{-2}$ $s^{-1}$)**
**for Each Year**

| Year | $g_{max}$ | $g_{min}$ |
|---|---|---|
| 2000 | 490 | 24 |
| 2001 | 400 | 18 |
| 2002 | 330 | 13 |

### Calculation of $FO_3$ and Models Comparison

Five 24-h cycles of ozone stomatal flux/ozone deposition flux estimations were compared with the measured ozone deposition fluxes (stomatal and nonstomatal deposition fluxes) for 2000 (Fig. 2): the mean $FO_3$ modeled from the experimental $g_s$, estimating the effective PAR ($FO_{3\_3\text{-}D}$) at each leaf height; the $FO_3$, using the multiplicative model ($FO_{3multiplicative}$), and three models for the estimation of ozone deposition fluxes based on the resistance model[4,7,11]. Only the resistance-based models of those described above that were significant for our data were applied (some models output data were completely abnormal for our data and had no sense at all, as there were too many outliers)(data not shown).

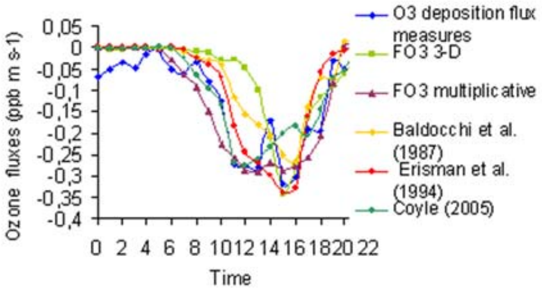

**FIGURE 2.** Ozone deposition fluxes and $FO_3$ for anthesis at 2000. Authors references indicate the resistance-type model used. Data compared are 1-h average values taken from 10-min average values.

The 2001 and 2002 cycles, for which the YPLANT model was included for a better effective PAR estimation at each leaf height, are shown in Figs. 3 and 4, respectively, except for the experimental ozone deposition measurements, which could not be achieved due to logistical problems.

## DISCUSSION

## Observational Data

### Stomatal Conductance and Leaf Level Grouping

All data for the 3 years were similar, except for the $g_s$ for 2000, when $g_s$ was quite higher than in 2001 and 2002 (see Tables 1 and 2). The more humid environmental conditions in 2000, with lower mean temperatures and higher RH values, can be invoked as a possible factor to explain this.

The results indicate that the wheat plant leaves can be grouped in homogeneous groups as a function of their $g_s$, usually the lowest leaves in height in a single group (with lower $g_s$) and the highest leaves in the other group (with higher $g_s$).

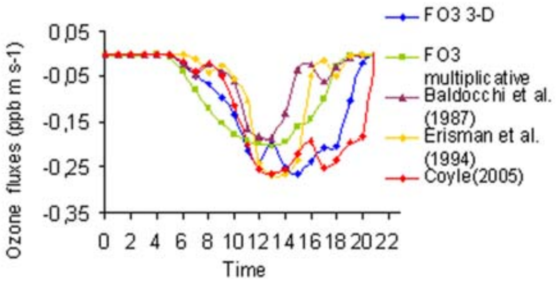

**FIGURE 3.** $FO_3$ for anthesis at 2001. Authors references indicate the resistance-type model used. Data compared are 1-h average values taken from 10-min average values.

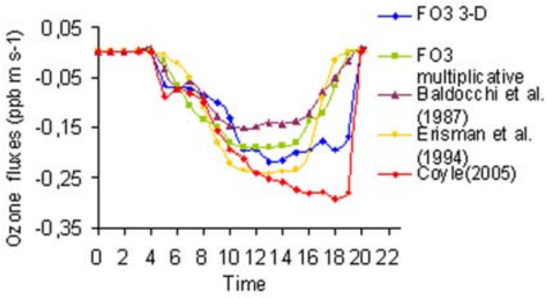

**FIGURE 4.** $FO_3$ for anthesis at 2002. Authors references indicate the resistance-type model used. Data compared are 1-h average values taken from 10-min average values.

## YPLANT Model Development and Models Comparison

### *Stomatal Conductance Modeling as a Function of Environmental Parameters*

PAR and VPD were the environmental parameters that proved to be the most significant among others as RH or temperature (data not shown: correlations were not as significant as with VPD). The positive coefficients would indicate direct correlations between the coefficient and $g_s$, whereas the negative coefficients would indicate inverse correlations. The VPD coefficient is negative, indicating the inverse modulation that VPD exerts over $g_s$.

### *Calculation of $FO_3$ and Model Comparison*

For the cycles in 2000, a maximum flux is seen around 15:00 h, except for the Coyle model[7], while minimum fluxes are observed at night. Visual analysis and the results from a regression analysis between the measured ozone deposition fluxes and the other models (Table 7) indicate that all the models performed

**TABLE 7**
**Regression Analysis for Measured Ozone Deposition Fluxes vs. Several Models of Ozone Deposition and Stomatal Ozone Fluxes, for 2000.**

| Model Compared to the Measured $O_3$ Deposition Fluxes | $R^2$ | *p* Value | n |
|---|---|---|---|
| $FO_{3\_3\text{-}D}$ | 0.53 | <0.05 | 24 |
| $FO_{3multiplicative}$ | 0.85 | <0.05 | 24 |
| Baldocchi et al.[4] | 0.82 | <0.05 | 24 |
| Erisman et al.[11] | 0.85 | <0.05 | 24 |
| Coyle[7] | 0.84 | <0.05 | 24 |

*Note:* Data compared are 1-h average values taken from 10-min average values.

in the same range and that all of them seemed to predict ozone deposition fluxes fairly well. The Erisman et al. resistance-based model[11] and the multiplicative model ($FO_{3multiplicative}$)[10] seemed to be the ones that best matched the ozone deposition flux cycle, although the multiplicative model underestimated fluxes during the midday hours. Nevertheless, despite their good performances (see Table 7), the Baldocchi et al.[4] and the Coyle[7] resistance-based models seemed to underestimate too much in the first half of the daily cycle and too much in the second half of the daily cycle, respectively. The $FO_{3\_3\text{-}D}$ seemed to estimate well during the midday hours, but it seemed to underestimate in the rest of the daily cycle. To avoid this problem, for 2001 and 2002, the YPLANT model was applied[28,39,40,41](see discussions above).

All the models suggest that stomatal conductance would account for the main percentage of ozone deposition fluxes, relying on the fact that cuticular resistance can be ignored and that soil resistance is assumed to be constant and not significant compared to stomatal resistance, contrary to the results of Gerosa et al.[14] and Altimir et al.[1] indicating that nonstomatal contributions to the ozone deposition fluxes may be up to 50%. The main difference between the way of estimating $FO_3$ based on the experimental $g_s$ and the effective PAR estimation ($FO_{3\_3\text{-}D}$) and all the other models, is that the former one estimates an overall weighted mean of the $FO_3$ for the canopy, whereas the multiplicative model and the resistance-based models estimate it at canopy level just considering that the main contribution to the total flux is due to the flag leaf[3].

The skewness shown in 2000 by the $FO_{3\_3\text{-}D}$ was no longer seen for these two years, 2001 and 2002, and the YPLANT improvement seemed to offer a better prediction of $FO_3$. To confirm this, a new regression analysis was performed for each year, comparing the $FO_{3\_3\text{-}D}$ vs. all the other models in order to test how good the fit was (Table 8). For all of the comparisons, the regressions were significant. Again, the Baldocchi et al. model[4] underestimated the ozone fluxes for both years, even though the fit was very good for 2002 ($R^2 = 0.71$). The Coyle model[7] showed the best correlations for both years, but for 2002, visual analysis revealed a large overestimation. The Erisman et al. model[11] and the multiplicative model ($FO_{3multiplicative}$)[10] fitted very similar to the $FO_{3\_3\text{-}D}$, although the multiplicative model seemed to be too dependent on $g_{max}$, since it is the main factor that determines $FO_{3multiplicative}$ (see Table 6), $FO_{3multiplicative}$ being directly correlated to $g_{max}$ and the highest $FO_{3multiplicative}$ being seen in 2000, when $g_{max}$ was maximum, and the lowest $FO_{3multiplicative}$ for 2002, when $g_{max}$ was minimum. The Erisman et al. model[11] thus seemed to be the resistance-based model that best estimated ozone deposition fluxes, although it seems to be the simplest one (see earlier discussion).

Accordingly, the new $FO_{3\_3\text{-}D}$ used, based on an effective PAR estimation at each leaf height with the YPLANT model, estimates $FO_3$ quite accurately, although further research is needed to obtain better and significant results demonstrating that, although not so important, the lower leaves also contribute to canopy $FO_3$.

**TABLE 8**
**Regression Analysis for $FO_{3\_3\text{-}D}$ vs. Several Ozone Deposition and Stomatal Ozone Flux Models, for 2001 and 2002**

| Model Compared to the 3-D $O_3$ Deposition Fluxes | 2001 | | | 2002 | | |
|---|---|---|---|---|---|---|
| | $R^2$ | *p* Value | n | $R^2$ | *p* Value | n |
| $FO_{3multiplicative}$ | 0.59 | <0.05 | 24 | 0.62 | <0.05 | 24 |
| Baldocchi et al.[4]) | 0.50 | <0.05 | 24 | 0.71 | <0.05 | 24 |
| Erisman et al.[11] | 0.59 | <0.05 | 24 | 0.62 | <0.05 | 24 |
| Coyle[7] | 0.82 | <0.05 | 24 | 0.96 | <0.05 | 24 |

*Note:* Data compared are 1-h average values taken from 10-min average values.

## CONCLUSIONS

Durum wheat (*Triticum durum* Desf. cv. Camacho) leaves can be organized in level groups as a function of their stomatal conductance to water vapor ($g_s$) homogeneity. Although this grouping depends on the plants' phenological and physiological stages, the usual pattern is that consecutive leaves in height may be grouped; i.e., in simple terms, higher leaves vs. lower leaves.

Environmental parameters are seen to be very important as conditioning factors for stomatal conductance: RH, temperature, SWP (indirectly, soil water content), and ambient ozone concentrations and, especially, PAR and VPD. The use of modeling programs such as YPLANT is shown to be suitable for modeling the canopy plant architecture in order to estimate the effective PAR at several heights of the plant canopy, considering intra- and interplant shading.

The method proposed for calculating ozone stomatal fluxes ($FO_{3\_3\text{-}D}$) from experimental $g_s$ data and modeling them as a function of certain environmental parameters in conjunction with the use of the YPLANT model suggests that architectural models are usable and that a further development of them is desirable, as they estimate the canopy $FO_{3\_3\text{-}D}$ integrating and not neglecting the contribution of the lower leaves with respect to the flag leaf, although its significance is not extremely high in this study. The fact that other resistance-type models, such as the Erisman et al.[11], afford better estimates of ozone stomatal fluxes probably is due to a need of a better adjustment and improvement of the plant architecture-based model. This method could be applicable for several plant phenological stages.

## ACKNOWLEDGMENTS

Acknowledgment is given to TheScientificWorldJOURNAL Editor responsible for handling this manuscript. Thanks are given to Dr. Agueda González Rodriguez (IMIDRA) for her collaboration in certain measurements, such as those for LAI, and her intermediation with her research institute in order to be able to make the experimental measurements at El Encin. I am very thankful as well to Dr. Fernando Valladares for his contribution as counselor in the management of the YPLANT model.
